# Shaping medical student’s understanding of and approach to rural practice through the undergraduate years: a longitudinal study

**DOI:** 10.1186/s12909-018-1229-8

**Published:** 2018-06-22

**Authors:** Robin A. Ray, Louise Young, Daniel Lindsay

**Affiliations:** 1 College of Medicine and Dentistry, James Cook University, Townsville, 4811 Australia; 2 College of Public Health, Medical and Veterinary Sciences, James Cook University, Townsville, 4811 Australia

**Keywords:** Medical education, Medical students, Rural clinical clerkship, Model of practice, Undergraduate, Career choice

## Abstract

**Background:**

Over the last two decades medical schools have increased rural practice learning opportunities for students in an effort to improve recruitment to the rural workforce. James Cook University’s (JCU) medical school was established in northern Australia in 2000 with a specific focus on meeting the health needs of people in rural and remote areas.

As part of a longitudinal study this paper explores the situational and motivational learning factors contributing to the development of JCU’s medical students’ understanding of and approaches to rural medical practice.

**Methods:**

After completing each consecutive, compulsory rural clinical placement in Year Two, Four and Six of their MBBS program, JCU medical students were asked to complete a survey about their rural learning experiences. The survey consisted of a combination of single choice, Guttman and Likert scales and open response questions. Data from two open response questions were coded and thematically analysed. Content analysis enabled the predominant value of each theme to be calculated.

**Results:**

Collation of the survey data revealed 680 answers to both questions resulting in 1322 comments for analysis. Nine themes were categorized into clinical practice issues and person issues. The evolution of scope of practice across the years, the importance of inspirational mentors, access to urban areas and a sense of community were key findings. Positive rural clinical placement experiences inclusive of supportive ongoing learning opportunities and rural community living contribute positively to medical students’ interest in future rural medical practice. However, the ability to work for periods of time in both rural and urban settings suggested a possible need for a new additional model of practice.

**Conclusion:**

Clear links between a sense of community and belonging both professionally and socially as well as combined rural-urban practice options were important factors in the education and development of future rural practitioners. Ways to establish and support practice models incorporating both rural and urban locations needs to be investigated.

**Electronic supplementary material:**

The online version of this article (10.1186/s12909-018-1229-8) contains supplementary material, which is available to authorized users.

## Background

In response to the recognized imbalance of available health care between urban and rural locations, medical schools have increased rural practice learning opportunities for students in an effort to improve recruitment and retention of the rural health workforce. While a rural background and family considerations have been identified as a significant factors for willingness to practice in a rural setting [1–3], repeated rural clinical placements during training may also be influential in the recruitment of rural doctors [4–6].

Close attention is being paid to the value of the placement itself and the varying time spent in rural and remote clinical placements for the development of rural practice intention among students across health professions [7, 8]. Early studies question the association between rural placements and future practice, suggesting the evidence is inconclusive [9, 10]. More recent work argues for the importance of the rural placement experience. Studies from Canada and Australia specifically identify the influence of clinical experiences and the availability of further training as contributors to intention to practice in rural and remote locations [11, 12]. Yet a critical review based on North American medical schools questioned whether the clinical experience motivated interest in rural practice or whether it reinforced a pre-existing interest [13]. Additionally, Tolhurst et al. [14] suggest that rural practice experiences can engender urban origin students’ interest towards rural practice.

While many studies have explored rural intention to practice at a single time point in medical training [15, 16], there is a lack of longitudinal data that follows students throughout their pre-registration medical training. This study was designed to understand the factors that develop and shape medical student’s understanding of and approach to rural practice as they progress through the undergraduate years.

### Setting

James Cook University’s medical school was established in northern Australia in 2000 with a specific focus on meeting the health needs of underserved populations. The student selection process purposively recruits applicants from rural and remote backgrounds and the academic and practical aspects of the curriculum, emphasis the health needs of rural, remote, tropical, Aboriginal and Torres Strait populations. Consequently, during the six years of the Bachelor of Medicine Bachelor of Surgery (MBBS) program all medical students undertake 20 weeks of compulsory rural and remote clinical placements, at intervals across the course; four weeks in Year Two and eight weeks in Years Four and Six. These placements are in part supported by funding from the Commonwealth Government Department of Health through the Rural Health Multidisciplinary Training (RHMT) program (formerly Rural Clinical Training and Support - RCTS) program. However, JCU’s rural clinical program involves all MBBS students not just the 25% of students required by the fore mentioned Government funding. Early JCU graduate outcomes data indicates that rural clinical experience during training can translate to rural practice [17].

## Aim

As part of a longitudinal cohort study of medical students’ evolving intention to practice rurally, this paper explores the situational and motivational factors that contribute to the formation of future rural practitioners, as expressed by students following their consecutive rural and remote clinical placement experiences.

## Methods

This study used surveys to ascertain students’ attitudes and perceptions of rural practice in relation to the learning opportunities available during a recent rural clinical placement.

### Sample

Medical students returning from their first rural or remote placement which occurs in Year Two, attend a debriefing session to share experiences and synthesise their learning. At the end of this session in 2012 and 2013 information about this study was presented to 188 and 193 students respectively, consent to participate across the course obtained, then some students completed the surveys. A similar process also occurred for students entering the study at Year Four in the years 2013 to 2015 (186, 173 and 187 students respectively) or continuing from their participation in Year 2. As these cohorts of students progressed through the program in the years 2015–2017, email reminders about the study were sent during the last week of their rural clinical placement and students were encouraged to complete either a hard copy or an on-line version of a survey in their final year, Year 6 (175, 179, 179 students respectively). This enabled two cohorts to be followed through three rural rotations (Year Two, Four and Six) and one cohort through two rotations (Years Four and Six).

### Tool

The surveys were developed from the results of a previous study of rural registrars and an examination of literature, then piloted by academics and students. Minor modifications were made before the survey was circulated. The survey tool used a combination of single choice, Guttman and Likert scales and open responses [18] formatted using SurveyMonkey® software (Additional file 1). The University allocated student numbers were used as identifiers to enable responses to be tracked and correlated across the data collection period from 2012 to 2017.

The survey asked questions about intent to practice rurally when beginning the course, current intent, projected intent after internship, influence of the recent placement on rural intentions, significant situations and motivational factors arising from recent placement, and potential barriers to rural practice. Survey questions were modified for each data collection point to reflect the evolving knowledge, attitudes and experience of the student. This paper focuses on data from the two open response questions: “Can you describe any significant situations on your rural placement that informed your ideas about future practice” and “What factors would motivate you to undertake rural practice?”

Ethical approval to conduct the study was granted by James Cook University Human Ethics Committee (H4551/H6096).

### Data analysis

For the purposes of this paper, the open responses to questions about significant situations and motivational factors arising from their clinical placements were analyzed by year level using a cross sectional approach to this longitudinal dataset. As not all medical students had participated in consecutive surveys thus reducing the availability of longitudinal data, grouping the data by year level increased the sample available for analysis.

Surveys were completed by 167 Year Two students (44% response rate), 300 Year Four students (55% response rate) and 213 Year Six students (40% response rate). Data from 12 anonymous students with no declared year level were excluded from analysis.

Data were entered into excel spreadsheets and descriptively coded using colour highlighting and memos. Codes were constantly compared and contrasted by two researchers, inductively developing and refining themes into categories, using consensus to resolve any discrepancies. Content analysis was applied to systematically quantify the underlying elements in each theme to gauge the predominant value of that theme [19]. Frequencies were counted as the number of times the comment was made. Percentages were calculated by dividing the frequency of particular comments by the number of participants per year level.

## Results

Collation of the survey data revealed 680 answers to both questions resulting in 1322 comments for analysis (some students recorded “no comment”). The length of responses ranged from three or four words such as “diverse ED cases” or “long hours, closer community” to more detailed responses like “Over whole rural placements they have become synonymous with being the best opportunity to practice clinical and procedural skills, as students are closely supervised and supported by teams.”

Nine themes arose from the data encompassing both clinical practice themes and personal themes which varied in frequency across their undergraduate years as medical students responded to repeated rural clinical practice experiences (see Table 1).

**Table 1:** Frequency of themes and subthemes across year levels

Theme	Subtheme	Year 2 *n* = 167		Year 4 *n* = 300		Year 6 *n* = 213	
		Frequency	Percentage	Frequency	Percentage	Frequency	Percentage
Scope of practice	Diversity/variety	74	44	55	18	43	20
	Opportunities/experience	19	11	90	30	5	2.5
	Experience/involvement	2	1	35	11.5	91	44
Working with doctors	Mentor/ Role model	34	13	84	21.6	51	15
	Teacher/supporter	8	8	42	14	42	21
Realities of rural practice	Limited resources/long hours	18	11	21	7	26	12
	Autonomy	4	2	21	7	22	11
	Satisfaction	25	7	21	7	18	9
	Rural-Urban practice	0	0	19	6.3	24	11
Teamwork	Participation in	0	0	28	9	48	23.5
	Relationships	12	7	35	11.5	45	22
Altruism	Workforce need	6	3.5	9	3	7	3.5
	Making a difference	26	15.5	24	8	18	9
	Commitment to rural	14	8	23	7.5	15	7.5
Future pathway	Job opportunities & Pathways	26	14	76	25	61	29
	Ongoing support network	3	2	20	6.5	41	20
Finance	Incentives	14	8	44	14.5	22	11
	Remuneration	8	8	54	18.5	34	16
Family considerations	Good place to raise children	11	6.5	20	6.5	19	9
	Partner employment/support	6	3.5	18	6	15	7.5
	Distance from family/city	10	6	22	7	27	12.5
Lifestyle	Work/life balance	22	13	71	23.5	48	24
	Sense of community	56	33.5	80	26.5	56	26
	Size and location	11	6.5	20	6.5	18	10
	Availability of facilities/activities	36	21.5	48	16	16	9

### Clinical practice themes

The scope of rural medical practice was a predominant theme across the dataset. While the diversity and variety in practice featured in comments after each clinical experience regardless of year level, there was also a progressive development in understanding of what scope of practice meant to students across the years. After the first rural placement in second year, excitement and interest generated by diversity and variety of practice was evident (44%). Whereas after the second rural placement that occurred in their first full time clinical year, comments were more about the opportunities for experiences (30%)*,* leading into hands on practice and fuller involvement in their final year (44%).

*Broader spectrum of practice, more exciting (Year 2).*

*Large scope of practice, broader range of skills (Year 4).*

*Get to see more variety in rural practice than in an urban centre (Year 4).*

*Variety of medicine, being given the responsibility, seeing patients on my own, feeling part of the team (Year 6).*

*Dealing with rare and difficulty cases in a small town setting that most students would not encounter in the cities (Year 6).*

This progression of students’ perception of scope of practice is closely linked with relationships between the student, the supervising doctor and other staff as well as the available learning opportunities. The positive influence of a mentor became more apparent as students progressed through their rural clinical rotations (respectively 8, 12, 16.5%). Comments made by fourth year students in their second rural rotation indicated that they were looking for role models as they began to formulate their image of medical practice.

*It’s hard not to be influenced by someone who you hold in high regard, who loves what they do and where they do it (Year 2).*

*Seeing and having time with great doctors has changed my perception of rural towns (Year 4).*

*Just being with and experiencing what rural generalists do day to day made me realise what I want to do (Year 4).*

*Doctors who were so welcoming and appreciative of the hard work you put in to help the team (Year 6).*

The negative influence of supervisors was also evident from the comments of four senior students citing staff attitudes that left them concerned about a future in rural practice.

*I will never return to [place] due to their attitude towards us, medical superintendents bullying students (Year 6).*

*SMOs[Senior medical officer] with unreal expectations, were quite rude and unprofessional in their conduct (Year 6).*

The importance of the doctor as teacher and need for support grew with each placement, recognised most by students in their final year (rising from 8% through 14 to 21%) as they approached more autonomous practice. Interestingly this is coupled with an increasing recognition of the need for a professional support network by 20% of final year students as opposed to only 6.5% of fourth year students.

*Working with the rural doctors, seeing the patients first then the doctor patient interaction (Year 4).*

*Opportunities to acquire and develop new skills (Year 4).*

*Being responsible for handling patients on my own and being a productive part of the team (Year 6).*

*Having excellent rural senior medical doctors who specialized as rural generalists (Year 6).*

*Having a supportive network in a rural location (Year 6).*

While job opportunities, further training and career pathways in rural areas became more important in later years (14, 25, 29% respectively), there was a slight decline in comments identifying a future commitment to rural practice (8, 7.5, 7.5%).

*Ability to do GP [general practitioner] work and hospital work with some specialist training (Year 6).*

*Training opportunities, support networks and professional development are all important (Year 6).*

Some students in fourth year (6.3%) introduced an alternative to full time rural practice suggesting that they would be more interested in rural practice if it could be incorporated into urban practice. Combining rural and urban practice either formally or as a regular locum was also proposed as a viable alternative model of care provision by several sixth year (11%) students.

*Practice a few days a month in rural or remote area, live predominantly in a regional city (Year 4).*

*Half the terms per year urban and half rural, the best of both worlds (Year 6).*

*Returning to rural as a permanent reliever 8 weeks at a time (Year 6).*

### Personal themes

Interestingly, the frequency of family considerations as a motivating factor was consistently low across the year levels in this cohort (6.5 and 3.5%, 6.5 and 6%, 9 and 7.5%). However, year level was a differential factor when considering the availability of facilities for entertainment, sport and retail which appear to be more important for second year students (21.5%) and less for final year students (9%). While size and location of the rural town trend towards being more of a motivating factor for final year students (6.5 to 10%) when compared with second and fourth year.

*Nice people, gym, shopping centre, internet, social activities (Year 4).*

*Size of town, services and education available, distance to major city (Year 6).*

The lifestyle perceived as available to rural practitioners was recognized by some students as working long hours with limited resources (11, 7, 12%). Alternatively, others perceived the lifestyle as somewhat idyllic increasing with their experience of the realities of rural life following their second and third rural placement (13, 23.5 and 24%). More specifically, feeling part of the community was more important for second years following their first rural placement (33.5%) than for the more experienced fourth (26.5%) and sixth year (26%) students.

*Opportunities to become part of the community, a strong community spirit (Year 2).*

*Enjoyed living in a rural community, the lifestyle, the work/life balance (Year 4).*

*I love the balance between rural life and rural practice (Year 6).*

## Discussion

The findings from our study suggest that positive rural clinical placement experiences inclusive of supportive ongoing learning opportunities and rural community living may contribute to medical students’ interest in future rural medical practice. Situational and motivating factors focused on clinical themes and personal themes, however these were not exclusive categories as student comments often incorporated both when expressing their views about situations that informed and motivated them concerning future rural medical practice. Our data suggested that clear links between a sense of community and belonging both professionally and socially were important factors in the formation and education of future rural practitioners, but alternatives to full time rural practice incorporating a shared rural-urban model are also needed.

A key finding from this study was the evolution of student’s perceptions of rural practice and the potential for harnessing the initial enthusiasm generated by the available scope of practice and skill building in later clinical placements. Additionally, the increased capacity for hands-on learning that grows as students progress through the course was evident when students in years four and six suggested that they had greater access to learning opportunities in these placements than other placements in urban hospitals. This is consistent with Daly et al. [15] where students in their final years valued the extended clinical learning opportunities. However, as noted in a few of our student comments and supported by Wilson et al. [20], negative interactions with staff especially in small teams such as those found in many rural locations was harmful to the identity formation of rural practitioners. On the other hand balancing the student’s desire for some autonomous practice with patient safety and the need for supervision may be effectively achieved by incorporating senior students into the multidisciplinary team, an important motivating factor identified by this cohort.

While it is recognised by students and the wider profession that rural practitioners are often required to work long hours and students create an extra impost on that workload [21], facilitating a positive, rural clinical experience is a significant factor for continuing to build the rural workforce. The importance of passionate role models and supportive mentors in this process cannot be overestimated. Students who commented specifically about rural doctors generally admired their capacity to manage a range of cases, interact personally and professionally with their patients and work with other team members, while still having time to mentor students. This was particularly important in the clinical years of the course (Years Four and Six) when students were beginning to shape their ideas for future practice. The time and energy devoted to student education also has implications for the support universities provide for rural doctors to enhance their educational skills enabling them to continue to provide these positive learning experiences [22].

A new student perspective expressed in our study was the need for an additional staffing model that enabled doctors to combine work in both rural and urban locations, not just as locums, but on a more permanent basis. Students described situations where they would be able to work for set periods in rural areas in combination with a practice in a regional city or capital city, more than the current outreach clinics [23] or flying specialist services [24]. A similar idea of a “dual track” approch to practice was also suggested by recent graduates in a Canadian study [25]. Additionally, in response to presenting this finding at the Association for Medical Education in Europe’s international conference (AMEE) in August 2017, audience discussion supported the notion of a combined model including a working example of this model of practice being provided by a Canadian doctor who works in one rural area each a month. While this additional, alternate “dual track” model would not necessarily integrate doctors into rural communities long term, it may be considered to provide a much needed service improving continuity of care for rural people living with chronic illness. More work is needed at the health service level and the community level in Australia to investigate the feasibility of structured urban-rural practice positions.

The eventuality of rural medical practice, expressed more clearly by students returning from their rural internship in their final undergraduate year, was associated with access to ongoing support networks and further training pathways. Connecting these students into a rural generalist training pathway during their undergraduate course may provide an assurance about the future career possibilities with opportunities to gain specialty qualifications [26].

Our study also highlights the importance of community life in rural and remote settings. For many second year students, small rural towns were a new experience. As students move through the course, the dynamics such as size and location become more important, a finding verified by McGrail et al.’s work [27]. Being connected with the community expressed through a sense of community, access to facilities, sporting and community groups and the relationship with patients enabling a continuity of care, were important factors motivating rural practice. However, the capacity of the community to accommodate family needs such as spousal work and schools as identified in other studies about rural intention [1, 2] was less evident among students in this cohort. This may be attributed to the predominately younger age of these students, many of whom began this course when they were between the ages of 17 to 19 years. The age of the students in the early years could also be a factor influencing their experience of learning in a rural location especially for those with an urban background for whom managing differences in the social and cultural realities of rural life was a new experience.

Enabling students to feel engaged with and comfortable in a rural community has implications for the preparatory learning activities provided in the medical curriculum at the university as well as rural mentors who go beyond the clinical environment to facilitate community connections [28]. However, learning is also about medical students contributing to patients’ wellbeing during the consultation ameliorating the disruption to the doctor patient relationship. Hunt et al. [29] suggest that the community should be engaged with students in the learning experience. Further research into rural practitioners and community perspectives of the broader issues, challenges and practicalities of mentoring medical students into rural and remote practice is required.

## Conclusions

Experiential and motivational factors that contribute to the formation of future rural medical practitioners consist of a combination of diversity in clinical practice and personal issues embedded in the social context of each community-based learning experience throughout this cohorts’ undergraduate education.

Inspirational mentors and teachers spread across the undergraduate years engaged students in the realities and of rural practice and provided a great environment for learning clinical medicine. Relationships and participation were important factors that could influence students either positively or negatively, especially in small healthcare teams found most often in rural and remote locations.

Supportive mentors, ways to maintain links between rural and urban practice locations and engagement with communities are critical factors in shaping rural practice intent within the available scope of practice and associated learning opportunities. However, further research that engages with communities, practitioners and healthcare systems in negotiating a formalized rural-urban a model of care may be useful.

## Additional file

Additional file 1: Survey for Year 4 students. (PDF 167 kb)

## Abbreviations

GP: General practitioner, doctor practicing family medicine
JCU: James Cook University
MBBS: Bachelor of Medicine Bachelor of Surgery
RCTS: Rural clinical training support
RHMT: Rural health multidisciplinary training program
SMO: Senior medical officer, doctor with or working towards specialist registration
