# Supplementary material for: Shaping medical student’s understanding of and approach to rural practice through the undergraduate years: a longitudinal study
Source: BMC Med Educ. 2018 Jun 22;18:147. doi: 10.1186/s12909-018-1229-8 (PMC6013947; doi:10.1186/s12909-018-1229-8)
Supplement: Supplementary file 1 — Survey for Year 4 students. (PDF 167 kb) [file 12909_2018_1229_MOESM1_ESM.pdf]

MED 4 Evolution of rural practice intentions

The evolution of rural practice intentions through undergraduate years to ...

This part of the quesionnaire requires you to select the answer most closely aligned to your opinion, or to provide a brief comment to summarise your response.

***1. Please write your student number here to enable correlation of your responses**

2. At that time (MED1), which specialty interested you most?

- general practice
- adult medicine
- adult surgery
- child and adolescent health
- reproductive and neonatal health
- aged care
- critical care
- mental health
- emergency medicine

Other (please specify)

3. When you began the course (in MED 1), where did you plan to work after graduation?

- Urban area
- Regional centre
- Rural town
- Remote location

4. Now that you have completed two rural rotations where do you think you would like to work?

- Urban area
- Regional centre
- Rural town
- Remote location

5. Has your recent rural placement experience influenced your future career choices?

- Not at all
- A little
- 50/50
- Definately

***6. Can you describe any significant situations on rural placement that informed your ideas about future practice?**

***7. What factors would motivate you to undertake rural practice?**

MED 4 Evolution of rural practice intentions

8. After you have completing your internship, how likely are you to practise in:

	definitely	possibly	not likely	not at all
urban area	◯	◯	◯	◯
regional centre	◯	◯	◯	◯
rural town	◯	◯	◯	◯
remote location	◯	◯	◯	◯

9. It is recognized that there are barriers to working in rural and remote locations. Please rate each potential barrier below between 0 (no barrier) to 5 (insurmountable challenges).

	0 - no barrier	1 - minor considerations	2 - issues to work through	3 - negotiable challenges	4 - difficult challenges	5 - insurmountable challenges
Distance from a capital city	◯	◯	◯	◯	◯	◯
Access to sport and recreation activities	◯	◯	◯	◯	◯	◯
Cost of living	◯	◯	◯	◯	◯	◯
Opportunities for entertainment and culture	◯	◯	◯	◯	◯	◯
Access to health services	◯	◯	◯	◯	◯	◯
Willingness of partner/spouse	◯	◯	◯	◯	◯	◯
Work for a partner/spouse	◯	◯	◯	◯	◯	◯
Appropriate educational opportunities for children	◯	◯	◯	◯	◯	◯
Availability of locums	◯	◯	◯	◯	◯	◯
Availability of incentives to work in rural areas	◯	◯	◯	◯	◯	◯
Availability of support for further education	◯	◯	◯	◯	◯	◯

Thank you
